# Risk of Post-Myocardial Infarction Pneumonia with Proton Pump Inhibitors, H2 Receptor Antagonists and Mucoprotective Agents: A Retrospective Nationwide Cohort Study

**DOI:** 10.3390/jpm12010078

**Published:** 2022-01-09

**Authors:** Jimin Jeon, Jinkwon Kim

**Affiliations:** Department of Neurology, Yongin Severance Hospital, Yonsei University College of Medicine, Yongin-si 16995, Korea; jmk50040@gmail.com

**Keywords:** myocardial infarction, H2 receptor antagonist, proton pump inhibitor, mucoprotective agent, pneumonia

## Abstract

Patients with myocardial infarction (MI) are at high risk of developing pneumonia. Proton pump inhibitors (PPI) and H2-receptor antagonists (H2RA) are commonly used acid-suppressive medications to the patients with MI for gastrointestinal (GI) protection, which may increase the risk for pneumonia. We evaluated whether PPI, H2RA, and mucoprotective agents without anti-acid properties increase the risk of post-MI pneumonia. We performed a retrospective cohort study based on the National Health Insurance Service—National Sample Cohort in Korea. The study included 3701 patients discharged with MI without prior history of pneumonia. During follow-up, treatments with PPI, H2RA, and mucoprotective agents were collected as time-dependent variables based on the prescription records. We performed multivariate time-dependent Cox regression analyses for the development of post-MI pneumonia. During the mean 4.85 ± 3.75 years follow-up, 999 participants developed pneumonia. In the multivariate analyses (adjusted hazard ratio; 95% confidence interval), the risk for pneumonia was significantly increased in treatment with PPI (2.25; 1.57–3.21) and H2RA (1.50; 1.16–1.93). Meanwhile, the risk for pneumonia was not increased in treatment with mucoprotective agents. When we evaluated GI bleeding event according to the medications as a secondary outcome analysis, mucoprotective agents were associated with increased GI bleeding risk, but PPI and H2RA were not. In the use of the GI medications in the treatment of patients with MI, the influence of these drugs on bleeding and pneumonia should be considered.

## 1. Introduction

Myocardial infarction (MI) is a critical disease with consequences in mortality and morbidity worldwide [1]. Patients with MI generally tend to be elderly and have high burden of comorbid diseases, malnutrition, medications, and stress, which make the patients more susceptible to infectious complications [2,3,4]. Pneumonia is one of the post-MI infections which increase the risk of death and the cost of care after MI [5]. Therefore, identifying underlying risk factors of post-MI pneumonia is an important step towards preventing the patient groups with MI at high susceptibility to pneumonia.

In the patients with MI, acid-suppressive drugs such as proton pump inhibitors (PPI) and H2-receptor antagonists (H2RA) are commonly used regarding the high gastrointestinal (GI) bleeding risk with regular use of aspirin or other antithrombotic medications as standard treatment to MI [6]. However, these acid-suppressive medications may be implicated in the increased risk of infection by attenuating of gastric acid, a major defense mechanism against infectious agents, which predispose patients to pneumonia [7]. Prior studies have consistently showed a positive association between acid-suppressive drugs and pneumonia [8,9,10,11]. However, there are few data on whether treatment with acid-suppressive medications increased the risk for pneumonia in post-MI patients. Along with PPI and H2RA, mucoprotective agents are a common anti-ulcerative medication with little or no anti-acid properties [12]. Mucoprotective agents without anti-acid properties may not increase the risk for pneumonia [9]. To evaluate the effects of acid-suppressive medications and mucoprotective agents on the risk of pneumonia in MI patients, we conducted a retrospective cohort study using a nationwide population-based health claims database in Korea. We also analyzed the relationship between use of the anti-ulcerative medications and the risk of GI bleeding in the MI patients.

## 2. Materials and Methods

### 2.1. Data Source

We carried out a retrospective cohort study conducted using the National Health Insurance Service-National Sample Cohort (NHIS-NSC), performed by the NHIS, a single national insurance provider in Korea [13]. The data were constructed with 1,025,340 participants who were random sampling stratified by sex, age, and household income, maintaining a sampling rate of approximately 2.2% of the entire Korean population in 2002. The NHIS-NSC datasets contain the participants’ entire health claim data between 2002–2015 including hospital visits, procedures, diagnosis, prescriptions and demographics information, and death statistics based on the International Statistical Classification of Diseases and Related Health Problems 10th Revision (ICD-10). The data of NHIS-NSC were fully anonymized. The Institutional Review Board of Yongin Severance Hospital (9-2020-0106) approved the study and waived the requirement for informed consent regarding retrospective analysis using anonymized data.

### 2.2. Study Participants and Outcome

**Figure 1:**
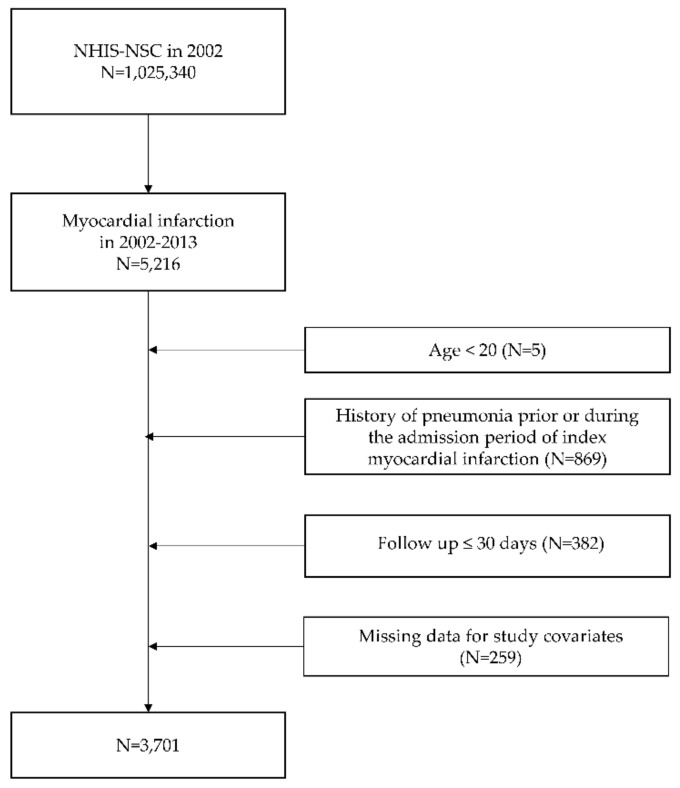
Flowchart of included patients. NHIS-NSC, National Health Insurance Service national sample cohort.

The study participants were patients aged >19 years who had admitted between 2002 and 2013 with a main diagnosis of MI (I21 in ICD-10) [14]. The primary outcome is defined as the development of pneumonia established based on the diagnostic code of ‘J10-J18’ after discharge [9,15,16]. To detect only patients with newly diagnosed pneumonia after MI as the outcome, patients who had been diagnosed with pneumonia prior to the discharge date of the index MI or who had less than a month of follow-up after discharge were excluded. A flow chart of the inclusion and exclusion criteria of participants is shown in Figure 1. From the date of discharge of index MI, the participants were followed up until the development of pneumonia, death, loss of eligibility for NHIS, or December 31, 2015 (whichever was earliest). As secondary outcomes, we evaluated the development of GI bleeding during follow-up. Development of GI bleeding events was defined as admission with the related diagnostic code of ‘K22.6, K25.0, K25.2, K25.4, K25.6, K26.0, K26.2, K26.4, K26.6, K27.0, K27.2, K27.4, K27.6, K28.0, K28.2, K28.4, K28.6, K29.0, K62.5, K92.0, K92.1, K92.2’ and receiving red blood cell transfusion during the admission [17].

### 2.3. Drug-Exposure Assessment

NHIS-NSC contains information on prescription drugs including drug name, drug code, date of prescription, daily dose, and period. We collected the prescription data of each patient for PPI (omeprazole, lansoprazole, ilaprazole, rabeprazole, pantoprazole, dexlansoprazole, and esomeprazole), H2RA (famotidine, ranitidine, lafutidine, cimetidine, roxatidine, and nizatidine), and mucoprotective agents (teprenone, rebamipide, ecabet, irsogladine, polaprezinc, sucralfate, misoprostol, and sofalcone). During follow-up period, exposures to PPI, H2RA, and mucoprotective agents were assessed every day after discharge date as time-dependent variables. For sub-analysis of PPI and H2RA intensity, PPI agents were further stratified into no use, low dose, or high dose by daily PPI dose. Low dose PPI were defined as up to ilaprazole 20 mg, omeprazole 20 mg, pantoprazole 40 mg, lansoprazole 30 mg, rabeprazole 20 mg, esomeprazole 30 mg, and dexlansoprazole 30 mg per day. Low dose H2RA were defined as up to cimetidine 800 mg, famotidine 40 mg, lafutidine 20 mg, nizatidine 300 mg, ranitidine 300 mg, and roxatidine 150 mg. The medications over the cut-off values of each agent per day were classified as a high dose PPI and high dose H2RA. The cut-off values were determined by the World Health Organization [18]. Treatment with antiplatelet and anticoagulant after MI was determined as exposure to antiplatelet and anticoagulant (oral and intravenous) within 30 days after MI admission.

### 2.4. Covariates

From the NHIS-NSC, we collected sex, age at MI admission, length of hospital stays at index MI, household income, treatment with antiplatelet and anticoagulant, and the presence of hypertension, diabetes mellitus, heart failure, stroke, chronic obstructive pulmonary disease, and prior history of GI bleeding as covariates potentially affecting the development of pneumonia. As a marker of severity and disability, length of hospital stays at index MI was classified into two groups (≤6 days, >6 days) according to median length of hospital stays following index MI. Hypertension (I10–15) [19], diabetes mellitus (E11–14) [20], heart failure (I50) [21], stroke (I60–64) [22] and chronic obstructive pulmonary disease (J42–44 (except for J43.0)) [23] were ascertained by the presence of diagnostic code before or during hospitalization for the index MI. Hypertension and diabetes mellitus were determined as relevant only if patients had received antihypertensive or antidiabetic medications at the time of diagnosis. Prior history of GI bleeding was defined as the presence of the related code before or at the discharge date of index MI.

### 2.5. Statistical Analyses

Continuous variables were described as mean ± standard deviation and categorical variables were described as numbers with percentages (%). For continuous variables, differences between groups were compared using independent *t*-test or analysis of variance test. For categorical variables, this comparison was made using chi-square test. As survival analyses, time-dependent Cox proportional hazard regression models were used to calculate hazard ratio (HR) and 95% confidence interval (CI) for the risk of pneumonia according to use of PPI, H2RA, and mucoprotective agents during follow-up periods, which were treated as time-dependent variables. Multivariate models were adjusted for sex, age, household income, length of hospital stays, and the presence of hypertension, diabetes mellitus, heart failure, stroke, chronic obstructive pulmonary disease, prior history of GI bleeding, treatment with antiplatelet and anticoagulant as time-fixed covariates. We additionally performed a sensitivity analysis according to PPI and H2RA intensity on the risk of pneumonia in MI patients. Linear trends between PPI/H2RA intensity and the risk of post MI pneumonia were evaluated by regarding the intensity groups as a continuous value (no use as 0, low dose as 1, and high dose as 2). As secondary outcome analysis, we evaluated the risk of GI bleeding events according to treatment with those agents during follow-up. The assumption of proportional hazards for PPI, H2RA, and mucoprotective agents in the Cox models were evaluated using a Grambsch-Therneau test of the scaled Schoenfeld residuals, which showed the proportional hazards assumption was satisfied. All statistical analyses were performed using the SAS 9.4 version (SAS Inc., Cary, NC, USA) and R software 3.4.4 version (The R Foundation for Statistical Computing, Vienna, Austria; http://www.R-project.org/ (accessed on 15 March 2021)). The statistically significant level for all tests was defined as two-sided *p*-value < 0.05.

## 3. Results

### 3.1. Baseline Characteristics

According to the inclusion and exclusion criteria, 3701 MI patients who were discharged without pneumonia were finally included (Figure 1). Baseline characteristics of the included patients are shown in Table 1. The median age was 62.01 ± 12.76 (mean ± standard deviation) years, and 2664 (71.98%) were male. The proportions of patients who received treatment with PPI, H2RA, and mucoprotective agents throughout 30 days after MI discharge were 12.46%, 21.32%, and 25.24% respectively.

### 3.2. Risk for Post-MI Pneumonia

During 4.85 ± 3.75 years of follow-up after index MI, 999 patients developed pneumonia (26.99%). To identify the independent risk factors for the development of post-MI pneumonia, we performed univariate and multivariate time-dependent Cox proportional hazard regression analyses (Table 2). In the multivariate model, use of PPI (adjusted HR; 95% CI, 2.25; 1.57–3.21, *p* < 0.001) and H2RA (adjusted HR; 95% CI, 1.50; 1.16–1.93, *p* = 0.002) were significantly associated with an increased risk for post-MI pneumonia. In contrast, we did not observe a significant association between use of mucoprotective agents and the risk for post-MI pneumonia (adjusted HR; 95% CI, 1.27; 0.98–1.64, *p* = 0.074). We additionally performed the risk of post-MI pneumonia according to PPI and H2RA intensity (Table S1). Compared with no PPI use, there was a significantly increased risk for post-MI pneumonia with low intensity of PPI. The risk for post-MI pneumonia increased with lower and higher dose of H2RA compared with no H2RA use.

### 3.3. Risk for Post-MI GI Bleeding

We performed analysis to assess the effect of those agents on the risk for GI bleeding among MI patients (Table 3). After index date of MI discharge, there were 100 patients (2.70%) who suffered GI bleeding event during follow-up. In the multivariate model for the GI bleeding after MI, we found that use of mucoprotective agents was associated with an increased risk for GI bleeding (adjusted HR; 95% CI, 2.92; 1.61–5.30, *p* < 0.001). There was no significant association between risk for GI bleeding and use of PPI and H2RA agents.

## 4. Discussion

This population-based cohort study investigated the risk of pneumonia according to the use of PPI, H2RA, and mucoprotective agents during the long-term follow-up period after MI. In the current study, the development of pneumonia was common after discharge from MI, wherein 27% of the included patients suffered from it. We documented that treatment with the acid-suppressive agents (PPI and H2RA) was significantly associated with an increased risk of post-MI pneumonia. On the other hand, use of mucoprotective agents was not associated with the risk of post-MI pneumonia.

Patients with prior cardiovascular disease including MI and stroke are frequently old and have concurrent illnesses, which predispose them to infectious disease [24,25]. With old age and a high burden of multiple comorbidities, patients with MI are likely to have physical deconditioning, cognitive impairment, derangement of hormonal system which may lead to an imbalance of anabolic–catabolic metabolism, resulting in malnutrition and immune suppression [26]. It has been well-established that concomitant diagnosis of prior cardiovascular disease is very common in patients with pneumonia [27,28]. Patients with comorbid cardiovascular disease are more likely have more severe pneumonia and long-term mortality after pneumonia [27,29,30]. Furthermore, pneumonia itself can increase risk for future development of cardiovascular event [31]. Regarding the high prevalence and adverse effect of pneumonia in the patients with MI, a better understanding of the pathophysiology may help to prevent the post-MI pneumonia, a potentially fatal complication and one of major health burden [28].

In general, MI patients need life-long antiplatelet therapy as secondary cardiovascular prevention. However, it is known that aspirin, the most widely used antiplatelet, is associated with a variety of GI side effects [5,32]. Along with aspirin, other antithrombotics are also involved in topical injury, depletion of prostaglandin from the gastric mucosa, which results in GI symptoms, epithelial damage in GI mucosa, and predisposition to bleeding [33,34,35]. For control and prevention of GI symptoms and bleeding, treatment with PPI or H2RA is widely used in patients with MI at the high-risk [36,37]. Despite the widespread use of PPI and H2RA, especially to patients with MI, there remains a concern that PPI and H2RA can increase the risk of pneumonia in patients [8,9,10,38]. PPI and H2RA agents protect GI by suppressing acid secretion, which attenuates the gastric acid barrier against pathogen invasion. There is much epidemiologic evidence supporting that concomitant use of PPI and H2RA increases the risk for oral, respiratory, and GI infections [39,40,41]. PPI undergoes principally hepatic metabolism by the cytochrome P4502C19 (CYP2C19), which is affected by genetic differences. A prior study reported that poor metabolizer, one of the genotypes variabilities of CYP2C19, had higher blood levels of PPI and higher intragastric pH, which may be linked to great risk for pneumonia development [42]. This might be particularly relevant for the Korean population which has a high incidence of poor metabolization. Regarding the consistent findings for the potential risk for pneumonia, it should be noted that unnecessarily prolonged use of the anti-acid medications can be harmful to the MI patients at high risk for pneumonia without proper indications. A recent coronavirus disease 2019 (COVID-19) study found that PPI usage increased the risk of severe outcomes (oxygen therapy, intensive care unit, invasive ventilation, or death) among COVID-19 patients [43]. Therefore, physicians need to be cautious in prescribing PPI to patients with COVID-19.

In this study, unlike PPI and H2RA, there was no significant association between use of mucoprotective agents and the risk of pneumonia. Mucoprotective agents are another class of gastroprotective agents by increasing prostaglandin levels, stimulating epidermal growth factor production, scavenging of hydroxyl radicals, and suppression of gastric mucosal inflammation rather than acid suppression [44,45,46]. Therefore, mucoprotective would be a practical option for patients who are at high risk of developing pneumonia.

However, in the secondary outcome analysis for post GI bleeding outcome, we found that the use of mucoprotective agents was associated with increased GI bleeding in MI patients. The paradoxical finding is expected because this is a retrospective observational study and mucoprotective agents are more likely to be prescribed to the patient groups considered at high risk of GI bleeding in clinical practice. We supposed that the unmeasured high GI bleeding risk in patients receiving mucoprotective agents led to more development of GI bleeding in the analysis; confounding effect rather than true effect of muco-protective agents inducing GI bleeding. Reducing mucosal injury with mucoprotective agents has been established in prior randomized trials [47,48]. The Korean cohort study found that misoprostol and rebamipide, along with PPI and H2RA, significantly reduced the risk of GI bleeding in nonsteroidal anti-inflammatory drug users for osteoarthritis or rheumatoid arthritis [49]. In the current study, use of other anti-ulcerative medications of PPI and H2RA did not have a significant association with post MI GI bleeding. These findings suggest that the GI protective effect of the mucopretective agent might be insufficient compared to the PPI and H2RA in the MI patients. Because PPI has been proved to be effective in prevention and treatment of upper GI bleeding, prescription of PPI is necessary to patients considered at high risk of GI bleeding and who have clear indications of PPI [50].

Based on the nation-wide health claims database, we performed a relatively long period of follow up (median 4.85 ± 3.75 years) for the development of pneumonia and identified the full drug prescriptions of the included patients. With the strength of the study, several potential limitations should be mentioned. First, with the limitation of retrospective observational design without interventions, we could not conclude the causal relationship between the medications and the risk of pneumonia. Further studies should be implemented to establish the effect of acid-suppressive agents and other gastroprotective agents on post-MI pneumonia. Second, we were unable to collect some genetic information and behavioral risk factors associated with susceptibility to pneumonia, such as smoking status and alcohol consumption due to the lack of data in the health claim database [51,52,53,54]. Although we identified whole medications by the prescription records of each patient in the NHIS database, there might be gap between the actual medications intake and prescriptions.

## 5. Conclusions

In conclusion, treatment with acid-suppressive agents such as PPI and H2RA is associated with an increased risk of post-MI pneumonia, whereas mucoprotective agents were not associated with the development of pneumonia. We did not find sufficient preventive effect of mucoprotective agents against GI bleeding after MI. When administering anti-ulcerative medications to MI patients at high risk of GI bleeding and pneumonia, clinicians should consider the underlying risks of each patient and the characteristics of the medications.

## Figures and Tables

**Table 1:** Baseline characteristics of included patients.

Variable	Value
Total number of patients	3701
Sex, male	2664 (71.98)
Age, years	62.01 ± 12.76
Hypertension	2833 (76.55)
Diabetes mellitus	1118 (30.21)
Heart failure	813 (21.97)
Stroke	657 (17.75)
Chronic obstructive pulmonary disease	903 (24.40)
Prior history of gastrointestinal bleeding	459 (12.40)
Household income	
low	1386 (37.45)
middle	1178 (31.83)
high	1137 (30.72)
Length of hospital stays	
≤6 days	2059 (55.63)
>6 days	1642 (44.37)
Treatment within 30 days after MI	
Antiplatelet	3598 (97.22)
Anticoagulant	3409 (92.11)

Data are number (%) or mean ± standard deviation. MI, myocardial infarction.

**Table 2:** Risk factors for the development of post-myocardial infarction pneumonia.

Variable	Unadjusted HR (95% CI)	*p* Value	Adjusted HR (95% CI)	*p* Value
Sex, male	0.63 (0.55–0.72)	<0.001	1.14 (0.99–1.32)	0.070
Age, years	1.06 (1.05–1.06)	<0.001	1.04 (1.03–1.05)	<0.001
Hypertension	1.73 (1.46–2.04)	<0.001	1.24 (1.04–1.47)	0.016
Diabetes mellitus	1.54 (1.35–1.75)	<0.001	1.33 (1.17–1.52)	<0.001
Heart failure	2.00 (1.74–2.30)	<0.001	1.39 (1.20–1.60)	<0.001
Chronic obstructive pulmonary disease	2.61 [2.29–2.97)	<0.001	1.66 (1.45–1.91)	<0.001
Stroke	2.40 [2.08–2.76)	<0.001	1.47 (1.27–1.71)	<0.001
Prior history of gastrointestinal bleeding	1.79 (1.51–2.14)	<0.001	1.36 (1.14–1.63)	<0.001
Household income				
low	1 (ref)	-	1 (ref)	-
middle	1.08 (0.93–1.25)	0.341	1.04 (0.89–1.21)	0.652
high	1.08 (0.93–1.26)	0.326	0.94 (0.80–1.09)	0.402
Length of hospital stays				
≤6 days	1 (ref)	-	1 (ref)	-
>6 days	1.39 (1.23–1.58)	<0.001	1.07 (0.94–1.22)	0.312
Treatment within 30 days after MI				
Antiplatelet	1.21 (0.84–1.75)	0.297	1.22 (0.81–1.83)	0.347
Anticoagulant	0.79 (0.65–0.97)	0.023	0.76 (0.60–0.94)	0.014
Anti-ulcerative medication				
Proton pump inhibitor	3.18 (2.24–4.53)	<0.001	2.25 (1.57–3.21)	<0.001
H2-receptor antagonist	2.06 (1.62–2.62)	<0.001	1.50 (1.16–1.93)	0.002
Mucoprotective agent	1.86 (1.47–2.37)	<0.001	1.27 (0.98–1.64)	0.074

Data were obtained from multivariate time-dependent Cox proportional hazard regression model. Adjustments were made to the variables listed in this table. Abbreviations: HR, hazard ratio; CI, confidence interval; MI, myocardial infarction.

**Table 3:** Risk for gastrointestinal bleeding according to the use of anti-ulcerative medications after myocardial infarction.

Variable	Unadjusted HR (95% CI)	*p* Value	Adjusted HR (95% CI)	*p* Value
Proton pump inhibitor	2.87 (1.04–7.94)	0.042	1.87 (0.67–5.22)	0.233
H2-receptor antagonist	1.90 (0.92–3.93)	0.082	1.07 (0.50–2.32)	0.858
Mucoprotective agent	3.53 (2.03–6.16)	<0.001	2.92 (1.61–5.30)	<0.001

Data were obtained from multivariate time-dependent Cox proportional hazard regression model for gastrointestinal bleeding after myocardial infarction. Adjustments were made to the same variables listed in the Table 2. Abbreviations: HR, hazard ratio; CI, confidence interval; MI, myocardial infarction.

## Data Availability

The dataset (NHIS-NSC) presented in this study is released by the National Health Insurance Sharing Service (http://nhiss.nhis.or.kr/bd/ab/bdaba021eng.do, accessed on 15 November 2021). Access to the dataset is available after submitting a completed application form, research proposal, and approval document from the institutional review board and receiving approval by the inquiry committee of research support in National Health Insurance Sharing Service.

## Supplementary Materials

The following supporting information can be downloaded at: https://www.mdpi.com/article/10.3390/jpm12010078/s1, Table S1: Effect of proton pump inhibitor and H2-receptor antagonist on the risk for post-myocardial infarction pneumonia according to dose intensity.
