# Supplementary material for: Risk of Post-Myocardial Infarction Pneumonia with Proton Pump Inhibitors, H2 Receptor Antagonists and Mucoprotective Agents: A Retrospective Nationwide Cohort Study
Source: J Pers Med. 2022 Jan 9;12(1):78. doi: 10.3390/jpm12010078 (PMC8778571; doi:10.3390/jpm12010078)
Supplement: Supplementary file 1 [file jpm-12-00078-s001.zip › jpm-1478033-supplementary.pdf]

Table S1. Effect of proton pump inhibitor and H2-receptor antagonist on the risk for post-myocardial infarction pneumonia according to dose intensity

Medication	Dose intensity	Adjusted HR [95% CI]	P value	P value for trend
Proton pump inhibitor	no use	Ref		
	low dose	2.61 [1.69-4.05]	<0.001	<.001
	high dose	1.75 [0.96-3.18]	0.069	
H2-receptor antagonist	no use	Ref		
	low dose	1.44 [1.05-1.96]	0.023	0.007
	high dose	1.63 [1.08-2.45]	0.019	

Data are derived by multivariate time-dependent Cox proportional hazard regression model adjusted for sex, age, household income, length of hospital stay, the presence of hypertension, diabetes mellitus, heart failure, stroke, and chronic obstructive pulmonary disease, prior history of gastrointestinal bleeding, and treatment with antiplatelet, anticoagulant within 30 days after myocardial infarction admission. Abbreviations: HR, hazard ratio; CI, confidence interval.
